# Postdischarge Glucocorticoid Use and Clinical Outcomes of Multisystem Inflammatory Syndrome in Children

**DOI:** 10.1001/jamanetworkopen.2022.41622

**Published:** 2022-11-11

**Authors:** Mary Beth F. Son, Laura Berbert, Cameron Young, Johnathan Dallas, Margaret Newhams, Sabrina Chen, Stacy P. Ardoin, Matthew L. Basiaga, Susan P. Canny, Hillary Crandall, Sanjeev Dhakal, Anita Dhanrajani, Anna Carmela P. Sagcal-Gironella, Charlotte V. Hobbs, Livie Huie, Karen James, Madelyn Jones, Susan Kim, Geraldina Lionetti, Melissa L. Mannion, Eyal Muscal, Sampath Prahalad, Grant S. Schulert, Kristen Sexson Tejtel, D. Sofia Villacis-Nunez, Eveline Y. Wu, Laura D. Zambrano, Angela P. Campbell, Manish M. Patel, Adrienne G. Randolph

**Affiliations:** 1 Division of Immunology, Boston Children’s Hospital, Boston, Massachusetts; 2 Department of Pediatrics, Harvard Medical School, Boston, Massachusetts; 3 Institute Centers for Clinical and Translational Research, Boston Children’s Hospital, Harvard Medical School, Boston, Massachusetts; 4 Department of Anesthesiology, Critical Care and Pain Medicine, Boston Children’s Hospital, Boston, Massachusetts; 5 Western Atlantic University School of Medicine, Freeport, Grand Bahama, Bahamas; 6 Rheumatology, Nationwide Children’s Hospital, Department of Pediatrics, Ohio State College of Medicine, Columbus; 7 Division of Pediatric Rheumatology, Department of Pediatric and Adolescent Medicine, Mayo Clinic, Rochester, Minnesota; 8 Division of Pediatric Rheumatology, Seattle Children’s Hospital, Department of Pediatrics, University of Washington, Seattle; 9 Division of Pediatric Critical Care, Department of Pediatrics, University of Utah, Primary Children’s Hospital, Salt Lake City; 10 Division of Rheumatology, Cincinnati Children’s Hospital Medical Center, Department of Pediatrics, University of Cincinnati College of Medicine, Cincinnati, Ohio; 11 Pediatric Rheumatology, Department of Pediatrics, University of Mississippi Medical Center, Jackson; 12 Department of Pediatrics, Hackensack Meridian School of Medicine, Hackensack, New Jersey; 13 Division of Rheumatology, Joseph M. Sanzari Children’s Hospital, Hackensack, New Jersey; 14 Division of Pediatric Infectious Disease, Department of Pediatrics, University of Mississippi Medical Center, Jackson; 15 Division of Pediatric Rheumatology, University of Alabama at Birmingham; 16 Division of Pediatric Rheumatology, Department of Pediatrics, University of Utah, Primary Children’s Hospital, Salt Lake City; 17 Pediatric Rheumatology, UCSF Benioff Children’s Hospital, Department of Pediatrics, University of California at San Francisco School of Medicine; 18 Division of Rheumatology, Texas Children’s Hospital, Department of Pediatrics, Baylor School of Medicine, Houston; 19 Children’s Healthcare of Atlanta, Department of Pediatrics, Emory University School of Medicine, Atlanta, Georgia; 20 Division of Cardiology, Texas Children’s Hospital, Department of Pediatrics, Baylor School of Medicine, Houston; 21 Division of Pediatric Rheumatology, UNC Children’s Hospital, Department of Pediatrics, University of North Carolina School of Medicine, Chapel Hill; 22 COVID-19 Response Team, Centers for Disease Control and Prevention, Atlanta, Georgia; 23 Public Health Service Commissioned Corps, Rockville, Maryland; 24 Departments of Pediatrics and Anesthesiology, Harvard Medical School, Boston, Massachusetts

## Abstract

**Importance:**

Minimal data are available regarding the postdischarge treatment of multisystem inflammatory syndrome in children (MIS-C).

**Objectives:**

To evaluate clinical characteristics associated with duration of postdischarge glucocorticoid use and assess postdischarge clinical course, laboratory test result trajectories, and adverse events in a multicenter cohort with MIS-C.

**Design, Setting, and Participants:**

This retrospective cohort study included patients with MIS-C hospitalized with severe illness and followed up for 3 months in an ambulatory setting. Patients younger than 21 years who were admitted between May 15, 2020, and May 31, 2021, at 13 US hospitals were included. Inclusion criteria were inpatient treatment comprising intravenous immunoglobulin, diagnosis of cardiovascular dysfunction (vasopressor requirement or left ventricular ejection fraction ≤55%), and availability of complete outpatient data for 3 months.

**Exposures:**

Glucocorticoid treatment.

**Main Outcomes and Measures:**

Main outcomes were patient characteristics associated with postdischarge glucocorticoid treatment, laboratory test result trajectories, and adverse events. Multivariable regression was used to evaluate factors associated with postdischarge weight gain (≥2 kg in 3 months) and hyperglycemia during illness.

**Results:**

Among 186 patients, the median age was 10.4 years (IQR, 6.7-14.2 years); most were male (107 [57.5%]), Black non-Hispanic (60 [32.3%]), and Hispanic or Latino (59 [31.7%]). Most children were critically ill (intensive care unit admission, 163 [87.6%]; vasopressor receipt, 134 [72.0%]) and received inpatient glucocorticoid treatment (178 [95.7%]). Most were discharged with continued glucocorticoid treatment (173 [93.0%]); median discharge dose was 42 mg/d (IQR, 30-60 mg/d) or 1.1 mg/kg/d (IQR, 0.7-1.7 mg/kg/d). Inpatient severity of illness was not associated with duration of postdischarge glucocorticoid treatment. Outpatient treatment duration varied (median, 23 days; IQR, 15-32 days). Time to normalization of C-reactive protein and ferritin levels was similar for glucocorticoid duration of less than 3 weeks vs 3 or more weeks. Readmission occurred in 7 patients (3.8%); none was for cardiovascular dysfunction. Hyperglycemia developed in 14 patients (8.1%). Seventy-five patients (43%) gained 2 kg or more after discharge (median 4.1 kg; IQR, 3.0-6.0 kg). Inpatient high-dose intravenous and oral glucocorticoid therapy was associated with postdischarge weight gain (adjusted odds ratio, 6.91; 95% CI, 1.92-24.91).

**Conclusions and Relevance:**

In this multicenter cohort of patients with MIS-C and cardiovascular dysfunction, postdischarge glucocorticoid treatment was often prolonged, but clinical outcomes were similar in patients prescribed shorter courses. Outpatient weight gain was common. Readmission was infrequent, with none for cardiovascular dysfunction. These findings suggest that strategies are needed to optimize postdischarge glucocorticoid courses for patients with MIS-C.

## Key Points

**Question:**

What are 3-month outcomes in patients with multisystem inflammatory syndrome in children (MIS-C), and what factors are associated with use of glucocorticoids following discharge?

**Findings:**

In this cohort study including 186 US children with MIS-C, severity of inpatient illness was not associated with duration of postdischarge glucocorticoid treatment; clinical outcomes were similar in patients prescribed shorter courses. Significant weight gain was common, but recurrent inflammation following hospital discharge was infrequent.

**Meaning:**

The findings of this study suggest that glucocorticoid tapers of less than 3 weeks are likely sufficient to treat MIS-C following discharge; shortening tapers is an important goal to avoid morbidity.

## Teaser

This cohort study examines 3-month outcomes of multisystem inflammatory syndrome in children (MIS-C) and factors associated with duration of glucocorticoid treatment for MIS-C after hospital discharge.

## Introduction

Multisystem inflammatory syndrome in children (MIS-C), a post-infectious inflammatory complication of COVID-19, is characterized by acute severe illness with prominent cardiovascular, gastrointestinal and mucocutaneous involvement.^1^ Clinicians extrapolated acute treatment strategies from Kawasaki disease (KD), as similar features of mucocutaneous and coronary artery involvement were noted.^2,3,4^ However, differences from KD were found, including older age and more frequent cardiovascular dysfunction in patients with MIS-C.^5,6^ Because MIS-C was a newly described syndrome, institutional and national treatment guidelines were published with the best evidence available as well as consideration for local practice^7,8,9^ and generally included intravenous immunoglobulin (IVIG)^10^ with or without glucocorticoids.^11^ Treatment regimens for patients with MIS-C during hospitalization have been studied, and initial treatment with IVIG plus glucocorticoids has been associated with improved short-term cardiovascular outcomes compared with use of IVIG alone.^12,13,14^ Glucocorticoid monotherapy may have benefit in some patients.^14,15^ Biologic agents, including anakinra, have also been used to treat MIS-C in children who are severely ill.^8,16^

The treatment of MIS-C following discharge has been less studied. Several weeks of oral glucocorticoid therapy have become routine at many centers, partly due to concerns about rebound inflammation associated with shorter treatment duration. The pathogenesis in MIS-C is unclear,^17^ obscuring indications and duration needed for postdischarge treatment. However, the acute course of MIS-C mimics other presumed postinfectious illnesses in childhood, including KD and immunoglobulin A vasculitis, which are treated with glucocorticoids.^11,18,19,20^ Although effective as anti-inflammatory medications, glucocorticoids have an undesirable adverse effect profile if administered daily to children for moderately prolonged periods, including deleterious effects on weight, mood, and glycemic control.^21^

The Overcoming COVID-19 network has enrolled hospitalized patients with MIS-C since March 2020. A collaboration between Overcoming COVID-19 and the Childhood Arthritis and Rheumatology Research Alliance (CARRA), a research network of pediatric rheumatologists in North America, was developed to expand data collection to MIS-C postdischarge management. In patients with MIS-C presenting with acute cardiovascular dysfunction and treated with IVIG, we aimed to identify patient characteristics associated with duration of postdischarge treatment and assess the postdischarge course.

## Methods

### Data Sources and Collaboration

We performed a retrospective cohort study using the Strengthening the Reporting of Observational Studies in Epidemiology (STROBE) reporting guideline.^22^ The Overcoming COVID-19 public health surveillance registry collected detailed clinical and medication data on US children and adolescents (<21 years of age) diagnosed with MIS-C via active surveillance at participating hospitals. Patients were identified at sites by intensive care unit (ICU) and subspecialty clinicians and through site reporting to state health departments. Trained staff at participating facilities abstracted medical records using a standard form and entered data into a web-enabled secure electronic database (REDCap; Vanderbilt University). CARRA investigators were surveyed for interest and if their sites participated in the Overcoming COVID-19 registry. An additional REDCap database was designed that linked the Overcoming COVID-19 inpatient data to the outpatient data abstracted by CARRA investigators. The surveillance protocol was reviewed by the Centers for Disease Control and Prevention (CDC) and the other participating institutions and was determined to be public health surveillance and not subject to informed consent requirements; this study was conducted in accordance with applicable federal laws and CDC policy.^23^ The surveillance protocol was approved by the central institutional review board at Boston Children’s Hospital under a waiver of informed consent.

### Study Population

We screened all consecutive patients in the Overcoming COVID-19 registry for the following characteristics: diagnosed with MIS-C between May 15, 2020, and May 31, 2021; laboratory confirmation of SARS-CoV-2 infection (positive SARS-CoV-2 real-time reverse transcriptase–polymerase chain reaction or antibody test result during hospitalization); cardiovascular dysfunction as evidenced by vasopressor support (dopamine, dobutamine, epinephrine, or norepinephrine) and/or depressed left ventricular ejection fraction (LVEF≤55%) on echocardiography during admission; and treatment with IVIG. Availability of complete outpatient data for 3 months was required for inclusion.

### Data and Study Measures

Data abstraction for the Overcoming COVID-19 registry included patient demographics, underlying medical conditions, presenting signs and symptoms, hospital course, laboratory test results, diagnostic studies, inpatient treatments, complications, and clinical outcomes. Race and ethnicity were abstracted from patient electronic medical records as reported by participating institutions and were included to evaluate for an association with outpatient treatment. The groups were categorized as Asian, non-Hispanic Black (hereafter Black), Hispanic or Latino, non-Hispanic White (hereafter White), and non-Hispanic other (hereafter other), which included the following categories: Alaska Native, American Indian, Native Hawaiian, Pacific Islander, and other. CARRA investigators extracted detailed data regarding outpatient medications and laboratory studies, readmissions within 90 days of the index admission, and noncardiac adverse events. Noncardiac adverse events included weight gain in the outpatient setting (defined as ≥2 kg in the 3 months after discharge), hyperglycemia (defined as ≥1 measurement of serum glucose ≥250 mg/dL during admission or at postdischarge follow-up [to convert to millimoles per liter, multiply by 0.0555]), the development of secondary autoimmunity (type 1 diabetes, hemolytic anemia, idiopathic thrombocytopenic purpura, or the development of antiphospholipid antibodies),^24,25,26,27^ documentation of infection during outpatient follow-up, development of transaminitis during illness (defined as 3 times the upper limit of normal for alanine transaminase or aspartate aminotransferase), gastrointestinal bleeding, or thromboembolism. Frequency of outpatient assessment varied across sites depending on clinic restrictions during the COVID-19 pandemic. Readmissions and weight gain were measured within 90 days after discharge. Outpatient weight gain was determined using the discharge weight and last weight recorded in the study period.

We assessed whether sociodemographic factors or clinical characteristics of inpatient illness, including days of fever, degree of organ involvement, KD features, admission to the ICU, length of hospital and ICU stays, or respiratory support (oxygen, noninvasive ventilation, and mechanical ventilation), were associated with duration of outpatient glucocorticoid treatment (<3 weeks vs ≥3 weeks). We also examined whether duration of postdischarge glucocorticoid treatment was associated with cardiac manifestations, including cardiovascular dysfunction, coronary artery aneurysms (defined as a *z* score of ≥2.5 of the left anterior descending coronary artery and/or right coronary artery),^2^ arrhythmias, and whether resolution of dysfunction (LVEF ≤55%) was documented before hospital discharge.

We assessed the association between the duration of glucocorticoid outpatient treatment and observed time to normalization of inflammatory markers (C-reactive protein [CRP] and ferritin) and weight gain in the outpatient setting. The threshold for elevated CRP level was set at greater than or equal to 1 mg/dL (to convert to milligrams per liter, multiply by 10) and ferritin level at greater than or equal to 250 ng/mL (to convert to micrograms per liter, multiply by 1) to account for different laboratory reference ranges. To assess trends in inflammatory markers in the outpatient setting, we selected laboratory measurements in the first 6 weeks following hospital discharge when most patients were reevaluated, modeled on routine management of KD.^2,28^

We examined inpatient glucocorticoid regimens, including specific glucocorticoid, dose, and route of administration (oral or intravenous). We defined high-dose oral glucocorticoid as either greater than or equal to 2 mg/kg/d or greater than or equal to 60 mg/d of prednisone and high-dose intravenous glucocorticoid as either greater than or equal to 10 mg/kg/d or greater than or equal to 500 mg/d of methylprednisolone.

We selected 2 binary outcomes for multivariable logistic regression: quantitative weight gain greater than or equal to 2 kg in the first 3 months after hospital discharge or development of hyperglycemia during illness. Associations of weight gain and hyperglycemia with clinical and treatment characteristics were assessed, including obesity (defined per CDC guidelines and body mass index),^29^ underlying medical conditions, type and dose of inpatient glucocorticoids, duration of inpatient oral glucocorticoid treatment, discharge dose (in 10-mg/d increments) and duration (in 1-week increments) of postdischarge glucocorticoid treatment, and indicators of severity of illness including hospital length of stay (in 1-day increments), ICU admission, vasopressor requirement, LVEF greater than or equal to 55%, and respiratory support.

### Statistical Analysis

Patient-level demographic and disease characteristics were summarized as frequency and proportion for categorical variables and median and IQR for continuous variables. Differences in continuous variables between glucocorticoid duration groups (<3 weeks and ≥3 weeks) were calculated using a Kruskal-Wallis test, and differences in categorical variables were determined using a Fisher exact test. Unadjusted logistic regression was used to identify variables associated with weight gain or hyperglycemia. An interaction between high-dose oral glucocorticoid treatment and high-dose intravenous glucocorticoid treatment was also considered (eTable 1 in Supplement 1). Based on these unadjusted results, variables with 2-sided, unpaired *P* ≤ .20 were considered for inclusion in a multivariable logistic regression model. We report adjusted and unadjusted odds ratios (ORs) and 95% CIs. Two-sided, unpaired *P* values ≤.05 were considered statistically significant. All analyses were conducted using R software, version 4.0.2 (R Project for Statistical Computing).

## Results

### Cohort and Inpatient Immunomodulatory Treatment

Data were collected from 186 patients (median age, 10.4 years [IQR, 6.7-14.2 years]; 79 female [42.5%]; 107 male [57.5%]) who met inclusion criteria at 13 volunteer sites participating in both the Overcoming COVID-19 registry and CARRA (eTable 2 in Supplement 1). Race and ethnicity categories were as follows: Asian, 3 (1.6%); Black, 60 (32.3%); Hispanic or Latino, 59 (31.7%); White, 40 (21.5%); other, 8 (4.3%); and unknown, 16 (8.6%). Most children were critically ill (ICU admission, 163 [87.6%]; vasopressor receipt, 134 [72.0%]) and received inpatient glucocorticoid treatment (178 [95.7%]). There was geographic variation across US regions (Northeast: 2 sites; South: 5 sites; Midwest: 3 sites; and West: 3 sites). Patients received IVIG on median hospital day 1 (IQR, 0-1), and 26 patients (14.0%) received a second dose of IVIG on median hospital day 2.5 (IQR, 2-3.8). Nearly all patients (178 [95.7%]) were treated with intravenous and/or oral glucocorticoids during admission. Forty-seven patients (25.3%) were treated with anakinra while hospitalized (median hospital day, 1 [IQR, 1-3]). The median maximum dose was 300 mg/d (IQR, 200-400 mg/d) given for a median of 8 days (IQR, 6-13 days) (eTable 3 in Supplement 1). Most patients (140 [80.9%]) were treated with aspirin while hospitalized.

### Patient Characteristics and Postdischarge Immunomodulatory Treatment

**Figure 1.**
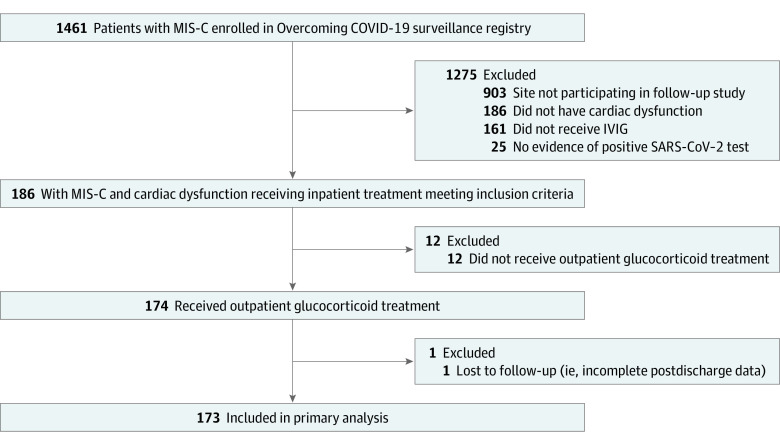
Diagram of Cohort Enrollment per Inclusion and Exclusion Criteria IVIG indicates intravenous immunoglobulin; MIS-C, multisystem inflammatory syndrome in children.

Of the 186 patients who received inpatient immunomodulation (Figure 1), most (173 [93.0%]) were discharged with outpatient immunomodulatory medications; of these patients, the median age was 10.2 years (IQR, 6.2-13.8 years), 76 (43.9%) were female, and 97 (56.1%) were male. Racial and ethnicity categories were as follows: Asian, 3 (1.7%); Black, 53 (30.6%); Hispanic or Latino, 54 (31.2%); White, 40 (23.1%), other, 8 (4.6%); and unknown, 15 (8.7%). A total of 56 children (32.4%) had obesity based on body mass index (defined as ≥95th percentile), and 44 (25.4%) were not previously healthy (Table 1). Outpatient glucocorticoid regimens varied by site (eFigure 1 in Supplement 1). Twelve patients did not receive outpatient immunomodulatory treatment and were excluded from the primary analysis (eTable 2 in Supplement 1); 1 patient was discharged with glucocorticoid therapy but had incomplete postdischarge data and was excluded (Figure 1).

**Table 1.** Demographic and Clinical Characteristics of Patients Hospitalized With Multisystem Inflammatory Syndrome in Children by Duration of Outpatient Glucocorticoid Treatment

Characteristic	Patients prescribed glucocorticoid treatment^a^			*P* value
	All (n = 173)	Duration <3 wk (n = 76)	Duration ≥3 wk (n = 97)	
Age, median (IQR), y	10.2 (6.2-13.8)	10.3 (6.9-12.5)	10 (5.6-14.1)	.89
Age, y				
<1	3 (1.7)	3 (3.9)	0	.09
1-4	28 (16.2)	8 (10.5)	20 (20.6)	
5-9	52 (30.1)	23 (30.3)	29 (29.9)	
10-14	63 (36.4)	32 (42.1)	31 (32.0)	
15-20	27 (15.6)	10 (13.2)	17 (17.5)	
Sex				
Female	76 (43.9)	37 (48.7)	39 (40.2)	.28
Male	97 (56.1)	39 (51.3)	58 (59.8)	
Race and ethnicity				
Asian	3 (1.7)	1 (1.3)	2 (2.1)	.004
Black, non-Hispanic	53 (30.6)	29 (38.2)	24 (24.7)	
Hispanic or Latino	54 (31.2)	29 (38.2)	25 (25.8)	
White, non-Hispanic	40 (23.1)	14 (18.4)	26 (26.8)	
Other, non-Hispanic^b^	8 (4.6)	0	8 (8.2)	
Unknown	15 (8.7)	3 (3.9)	12 (12.4)	
BMI-based obesity^c^	56 (32.4)	21 (27.6)	35 (36.1)	.26
Not previously healthy^d^	44 (25.4)	14 (18.7)	30 (30.9)	.08
Organ systems involved, No.				
≤4	59 (34.1)	25 (32.9)	34 (35.1)	.91
≥5	114 (65.9)	51 (67.1)	63 (64.9)	
Met criteria for incomplete or complete Kawasaki disease	59 (34.1)	23 (30.3)	36 (37.1)	.42
Duration of fever, median (IQR) [range], d (n = 153)	5 (4-6) [0-23]	5 (4-6) [2-13]	5 (4-6) [0-23]	.62
ICU admission	151 (87.3)	69 (90.8)	82 (84.5)	.26
Length of ICU stay, median (IQR) [range], d (n = 151)	3 (2-5) [0-26]	3 (2-5) [0-26]	3(2-6) [0-24]	.44
Length of hospital stay, median (IQR) [range], d	7 (5-9) [1-36]	6 (4-8) [3-32]	7 (5-10) [1-36]	.06
Supplemental oxygen	123 (71.1)	55 (72.4)	68 (70.1)	.73
Mechanical ventilation	26 (15.0)	12 (15.8)	14 (14.4)	.83
Vasopressor requirement	125 (72.3)	57 (75.0)	68 (70.1)	.50
ECMO	4 (2.3)	2 (2.6)	2 (2.1)	>.99

Abbreviations: BMI, body mass index; ECMO, extracorporeal membrane oxygenation; ICU, intensive care unit.

^a^ Data are presented as number (percentage) of patients unless otherwise indicated.

^b^ Included Alaska Native, American Indian, Native Hawaiian, Pacific Islander, and other.

^c^ Obesity was defined as a BMI equal to or greater than the 95th percentile.

^d^ Not previously healthy was defined as the presence of reported underlying conditions (respiratory, cardiovascular, neurologic, oncologic, immunosuppressive, rheumatologic or autoimmune, hematologic, kidney or urologic, gastrointestinal or hepatic, endocrine, or metabolic [including obesity]); long-term ventilation or oxygen support; and use of prescription diuretics, bronchodilators, glucocorticoids, statins, immunosuppressive drugs, or chemotherapy for any condition.

Dosages of postdischarge immunomodulatory treatments are detailed in Table 2. Patients prescribed outpatient glucocorticoid therapy had a median course of 23 days (IQR, 15-32 days) with a range of 1 to 107 days. The median discharge dose was 1.1 mg/kg/d (IQR, 0.7-1.7 mg/kg/d) or 42 mg/d (IQR, 30-60 mg/d).

**Table 2.** Outpatient Immunomodulatory Medication Regimens for Patients With Multisystem Inflammatory Syndrome in Children

Treatment	Median (IQR) [range]
Oral glucocorticoids (n = 173)^a^	
Prescription length, d (n = 170)	23 (15-32) [1-107]
Discharge dose, mg/kg/d (n = 170)	1.1 (0.7-1.7) [0.1-2.4]
Discharge dose, mg/d (n = 173)	42.4 (30-60) [1.2-130.2]
Anakinra (n = 8)	
Prescription length, d	14 (7.8-17.2) [1-35]
Discharge dose, mg/d	150 (100-200) [25-400]
Tapered to every other day before discontinuation, No. (%)	4 (50)

^a^ Prednisone and prednisolone.

Between patients discharged with less than 3 weeks (76 [43.9%]) vs 3 or more weeks (97 [56.1%]) of outpatient glucocorticoid treatment, there was no significant difference in age, sex, or clinical characteristics, including days of fever at admission, admission to ICU, length of ICU or hospital stays, respiratory support, vasopressor treatment, or use of extracorporeal membrane oxygenation (Table 1). Black and Hispanic or Latino patients were more likely to receive shorter courses of outpatient glucocorticoids.

No significant differences in cardiac characteristics, including cardiovascular dysfunction, coronary artery aneurysms, and arrhythmias, were observed between patients who received outpatient glucocorticoid treatment of less than 3 weeks vs those treated for 3 or more weeks (Table 3). Of note, 76.0% (98 of 129) of patients with LVEF less than or equal to 55% during admission had resolution of depressed cardiac function on echocardiography before hospital discharge. Among the 31 patients without documented resolution of depressed function before discharge, 28 (90.3%) were followed up by the cardiology service in the outpatient setting; median time to resolution of LVEF less than or equal to 55% on echocardiography was 13 days (IQR, 8-15 days) after discharge.

**Table 3.** Cardiac and Noncardiac Outcomes and Duration of Outpatient Glucocorticoid Therapy Among Patients With Multisystem Inflammatory Syndrome in Children

Variables	Patients prescribed outpatient glucocorticoids			*P* value
	Total (n = 173)	Duration <3 wk (n = 76)	Duration ≥3 wk (n = 97)	
Cardiac characteristics				
Vasopressor support, No. (%)	116 (67.1)	52 (68.4)	64 (66.0)	.75
Length of vasopressor support, median (IQR) [range], d^a^	1 (0-2) [0-7]	1 (0-2) [0-6]	1 (0-2.5) [0-7]	.61
LVEF ≤55% during admission, No. (%)	129 (74.6)	54 (71.1)	75 (77.3)	.38
Impaired LVEF resolved by discharge, No./total No. (%)	98/129 (76.0)	43/54 (79.6)	55/75 (73.3)	.53
Maximum *z* score ≥2.5 of LAD or RCA on inpatient echocardiography, No./total No. (%)	35/155 (22.6)	12/68 (17.6)	23/87 (26.4)	.25
Arrhythmias^b^	6 (3.5)	3 (3.9)	3 (3.1)	>.99
Laboratory outcomes, median (IQR) [range]				
Time until normalization of CRP since peak inpatient value (≤1 mg/dL), d^c^	12 (8-19) [4-128]	13 (8.3-21.8) [4-128]	11 (8-19) [4-59]	.38
Time until normalization of ferritin since peak inpatient value (≤250 ng/mL), d^d^	14 (5-25) [0-188]	13 (4-23.5) [0-188]	16 (6.3-25.8) [0-100]	.30
Adverse events, No. (%)				
Weight gain ≥2 kg at outpatient follow-up	75 (43.4)	29 (38.2)	46 (47.4)	.28
Hyperglycemia during illness^e^	14 (8.1)	7 (9.2)	7 (7.2)	.78

Abbreviations: CRP, C-reactive protein; LAD, left anterior descending coronary artery; LVEF, left ventricular ejection fraction; RCA, right coronary artery.

SI conversion: To convert CRP to milligrams per liter, multiply by 10; ferritin to micrograms per liter, multiply by 1.

^a^ Available for 171 patients (duration <3 wk, n = 76; duration ≥3 wk, n = 95).

^b^ First-degree or higher atrioventricular block, ST segment changes, prolonged QTc interval.

^c^ C-reactive protein level normalization documented in 150 patients (duration <3 wk, n = 65; duration ≥3 wk, n = 85).

^d^ Ferritin level normalization documented in 133 patients (duration <3 wk, n = 55; duration ≥3 wk, n = 78).

^e^ Hyperglycemia denotes 1 or more measurement of serum glucose greater than or equal to 250 mg/dL (to convert to millimoles per liter, multiply by 0.0555) during admission or postdischarge follow-up.

### Postdischarge Laboratory Trajectories

Duration of postdischarge glucocorticoid treatment was not significantly associated with a difference in time to normalization of CRP or ferritin levels (Table 3). Furthermore, the CRP and ferritin values decreased with infrequent rebound inflammation in the 6 weeks following discharge regardless of glucocorticoid treatment duration (eFigure 2 in Supplement 1).

### Adverse Events

Readmission was infrequent, with only 7 patients (3.8%) requiring readmission within 90 days of the index admission for MIS-C; none were readmitted for cardiovascular dysfunction. No patient required readmission for recurrent features of KD, increasing laboratory markers of cardiac injury (troponin and brain natriuretic protein levels), or findings on echocardiography, including no readmissions for worsening coronary artery dilation, left ventricular function, or arrhythmia.

Three patients (1.6%) were readmitted with symptoms possibly consistent with MIS-C, including fever and gastrointestinal symptoms (abdominal pain, nausea, and vomiting) in 2 patients and altered mental status in 1 patient. One patient (0.5%) required readmission for cellulitis at a surgical site. Three patients (1.6%) were readmitted for reasons deemed as other by the clinician, including dizziness and syncope with gastrointestinal bleeding (n = 1), secondary hypertension (n = 1), and glucocorticoid-associated hyperglycemia (n = 1). The median hospital stay for readmission was 3 days (range, 2-11 days).

Thirty-four patients (18.3%) developed transaminitis during the course of their MIS-C illness, with resolution in 24 patients before hospital discharge. Eight patients had persistently elevated transaminase levels for 4 or more weeks.

Other adverse events in the 90-day period following hospital discharge were uncommon. Two patients (1.1%) developed gastrointestinal bleeding after discharge. No patient developed thromboembolism after discharge. One patient (0.5%) was diagnosed with type 1 diabetes.

Among patients with weight gain greater than or equal to 2 kg (75 of 173 [43.4%]), the median increase in weight was 4.1 kg (IQR, 3.0-6.0 kg); duration of glucocorticoid treatment was not associated with weight gain (Table 3). Fourteen patients (8.1%) developed hyperglycemia during the course of their illness; 11 patients required inpatient insulin therapy with a median duration of 3 days (IQR, 3-20 days; range, 1-325 days).

### Associations of Weight Gain and Hyperglycemia With Patient and Treatment Characteristics

**Figure 2.**
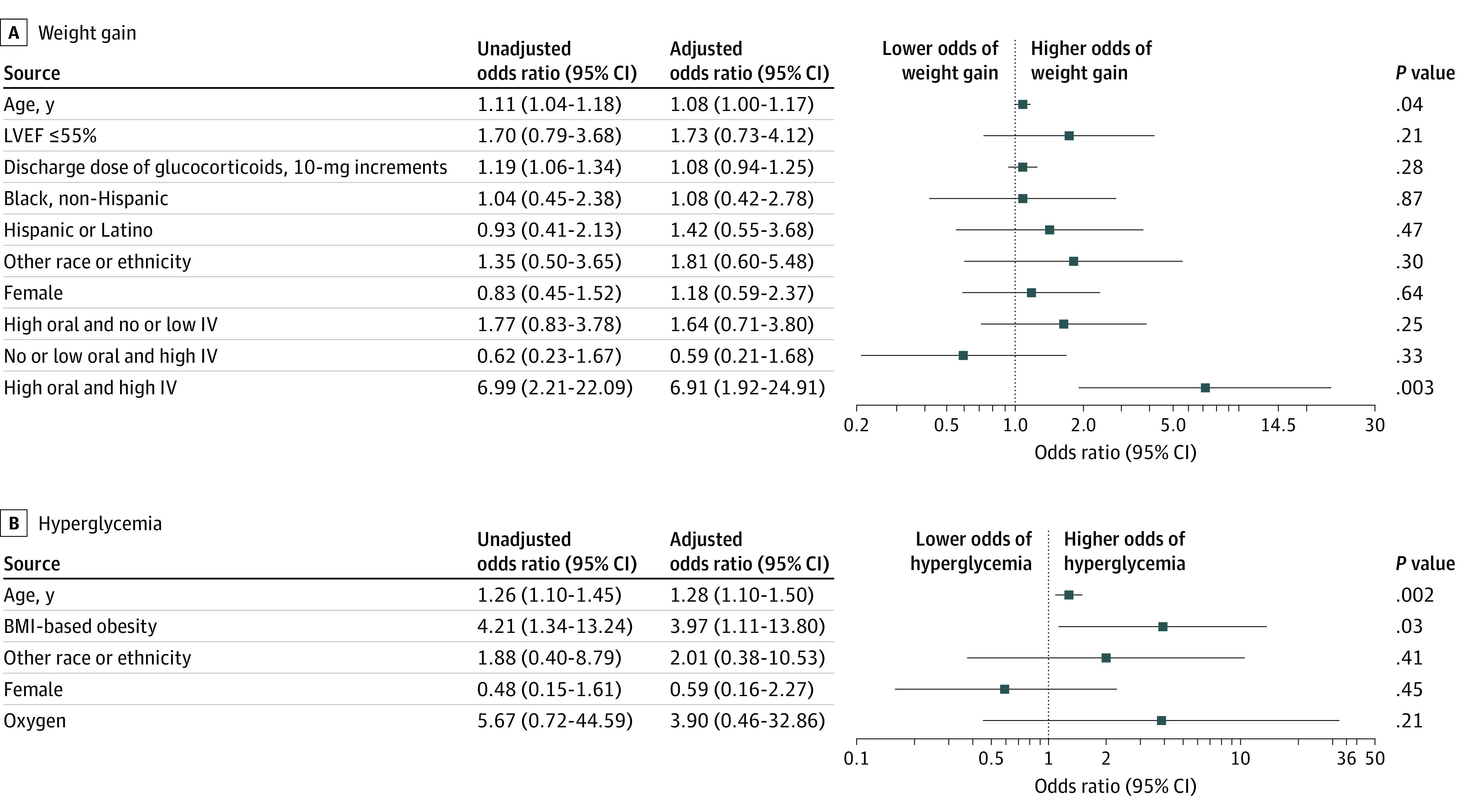
Analyses of Weight Gain and Hyperglycemia in Patients With Multisystem Inflammatory Syndrome in Children A, Unadjusted and adjusted analyses of factors associated with weight gain (≥2 kg) in the 3 months after discharge. The reference category for left ventricular ejection fraction (LVEF) was greater than 55%; discharge dose of glucocorticoids, 10-mg increment; race and ethnicity, non-Hispanic White; female, male; and treatment regimens, no or low-dose oral and intravenous glucocorticoid treatment. B, Unadjusted and adjusted analyses of factors associated with hyperglycemia during illness. The reference category for body mass index (BMI)–based obesity was no obesity; other race or ethnicity, non-Hispanic White; female, male; and supplemental oxygen; none. A and B, Other race and ethnicity included Alaska Native, Native American, Native Hawaiian, Pacific Islander, and other. IV indicates intravenous.

Multivariable modeling showed that outpatient weight gain was associated with inpatient high-dose glucocorticoid treatment regimen. Specifically, patients receiving inpatient high-dose intravenous glucocorticoids followed by high-dose oral glucocorticoids (as opposed to receiving high-dose intravenous glucocorticoids with low-dose oral glucocorticoids or low-dose intravenous glucocorticoids with high-dose oral glucocorticoids during hospitalization) were more likely to gain 2 kg or more after discharge (adjusted odds ratio [aOR], 6.91; 95% CI, 1.92-24.91; *P* = .003) (Figure 2A). Obesity (aOR, 3.97; 95% CI, 1.11-13.80; *P* = .03) and age (aOR, 1.28; 95% CI, 1.10-1.50; *P* = .002) (Figure 2B) were associated with hyperglycemia during illness in multivariable modeling.

## Discussion

In a multicenter cohort of patients with MIS-C with severe inpatient illness, duration of postdischarge glucocorticoid treatment varied across sites, but longer duration (≥3 weeks) was not associated with severity of illness or with better clinical outcomes. Inflammatory markers decreased in the postdischarge period, and rebound inflammation was infrequent, including in patients treated with glucocorticoids for shorter durations. Readmission was uncommon (3.8%), with no patients readmitted with cardiovascular dysfunction. Among patients who gained 2 kg or more, median weight gain was 4.1 kg in the outpatient setting and was a commonly documented adverse event in this population (43.4%). Significant hyperglycemia, a glucocorticoid-related adverse effect, occurred in 1 in 12 patients during acute illness or outpatient follow-up.

Initially during the COVID-19 pandemic, little was known about the etiology, treatment, or outcomes of MIS-C. Subsequently, it became more clear that the acute severe cardiovascular symptoms resolve fairly quickly with treatment,^30^ and effective inpatient treatments have been studied^12,13,14^ and recommended by national and international committees.^8,31^ Questions regarding longer-term outcomes, such as myocardial fibrosis, are under investigation.^28^ However, little has been studied regarding postdischarge treatment of MIS-C. In the absence of published data, many clinicians have opted for prolonged glucocorticoid tapers for ambulatory patients with MIS-C because there were concerns in the early phase of the pandemic regarding rebound inflammation with faster tapers. We did not identify substantial rebound inflammation in this population, as measured by repeated laboratory studies or clinical indicators of cardiovascular dysfunction and readmissions. Of importance, patients with shorter glucocorticoid tapers fared as well as those using longer tapers. These findings suggest that shorter outpatient glucocorticoid tapers (<3 weeks) are likely sufficient for patients with MIS-C with cardiovascular dysfunction.

Some features of MIS-C are phenotypically similar to those of KD, leading to treatment with IVIG and glucocorticoids. The RAISE trial was a landmark study of glucocorticoid treatment in high-risk patients with KD who were treated with inpatient intravenous glucocorticoids (2 mg/kg/d) and discharged with oral glucocorticoids tapered over 15 days.^11^ Glucocorticoid tapers in the cohort in the present study were longer, with a median of 23 days, which is notable in the context of weight gain and hyperglycemia.

When adjusting for illness severity indicators as well as discharge dose and duration of outpatient glucocorticoids, the combination of inpatient high-dose intravenous glucocorticoids followed by inpatient high-dose oral glucocorticoids was associated with weight gain in the outpatient setting. It is possible that randomized clinical trial data may become available to guide inpatient glucocorticoid treatment in the future, although that is unlikely to be the case for outpatient glucocorticoid regimens.^32^ Creation and implementation of consensus treatment plans^33^ with varying doses and duration of outpatient glucocorticoid therapy may provide observational data to determine optimal outpatient glucocorticoid regimens going forward. Of note, future research will likely be impacted by the incidence and severity of MIS-C with specific SARS-CoV-2 variants.^34,35,36^

Hyperglycemia was documented in this study. Stress hyperglycemia is well described in pediatric ICU patients, and nearly 90% of patients in the present cohort required ICU admission.^37^ In addition, obesity is associated with pediatric hyperglycemia,^38^ and obesity has been documented in up to one-third of cohorts with MIS-C across studies.^1,39,40^ In this study, obesity was associated with hyperglycemia, which may help identify a high-risk population for glucocorticoid treatment for MIS-C.

### Limitations

Our study has limitations. Although, to our knowledge, this is the largest study to date of outpatient therapy in patients with MIS-C who presented with cardiovascular involvement, it did not include all US centers and was not international, limiting generalizability. This was an observational study, which prevents ascribing causality in our findings. The cohort was enrolled before the emergence of the Delta and Omicron SARS-CoV-2 variants, which may have impacted MIS-C outcomes. Weight gain was likely multifactorial, and other unmeasured contributors, including restrictions in physical activity due to suspected or confirmed myocarditis and restricted activities due to school and activity closures, may have been associated with weight gain. Furthermore, the clinical implications of the weight gain observed in this study are unknown. Access to medical care differed across the regions of the US during the pandemic, which likely impacted collection of laboratory study results and may have led to prolonged glucocorticoid courses. In addition, sites that participated in our study demonstrated reasonable variation in treatment, and unmeasurable factors at sites could have impacted therapeutic decisions.

## Conclusions

In this study, we found that patients with MIS-C and cardiovascular dysfunction in a multicenter US cohort were frequently discharged with prolonged glucocorticoid tapers and had favorable clinical courses without rebound inflammation or recurrent cardiovascular dysfunction. Nearly half the patients gained a substantial amount of weight in the outpatient setting—a notable finding because underlying obesity has been consistently reported in up to one-third of patients with MIS-C. Although further study is required to determine optimal treatment of MIS-C, the findings of this study suggest that high inpatient glucocorticoid doses are associated with increased risk of complications and that prolonged outpatient courses should be avoided.

## Notes

Supplement 1. **eTable 1.** Glucocorticoid Inpatient Regimens and Outcomes for Patients Treated with Postdischarge Glucocorticoids **eTable 2.** Demographic and Clinical Characteristics of Patients Based on Outpatient Immunomodulatory Treatment **eTable 3.** Inpatient Immunomodulatory Treatment Regimens **eFigure 1.** Distribution of Patients Across Participating Centers and According to Duration of Glucocorticoid Outpatient Regimen **eFigure 2.** C-Reactive Protein (CRP) Measurements per Patient and Ferritin Measurements per Patient, According to Duration of Outpatient Glucocorticoid Regimen, in the Six Weeks Following Hospital Discharge

Supplement 2.
Overcoming COVID-19 Investigators

## References

[1] Feldstein LR, Rose EB, Horwitz SM, ; Overcoming COVID-19 Investigators; CDC COVID-19 Response Team. Multisystem Inflammatory syndrome in US children and adolescents. N Engl J Med. 2020;383(4):334-346. doi:10.1056/NEJMoa2021680 32598831 PMC7346765

[2] McCrindle BW, Rowley AH, Newburger JW, ; American Heart Association Rheumatic Fever, Endocarditis, and Kawasaki Disease Committee of the Council on Cardiovascular Disease in the Young; Council on Cardiovascular and Stroke Nursing; Council on Cardiovascular Surgery and Anesthesia; and Council on Epidemiology and Prevention. Diagnosis, treatment, and long-term management of Kawasaki disease: a scientific statement for health professionals from the American Heart Association. Circulation. 2017;135(17):e927-e999. doi:10.1161/CIR.0000000000000484 28356445

[3] Verdoni L, Mazza A, Gervasoni A, . An outbreak of severe Kawasaki-like disease at the Italian epicentre of the SARS-CoV-2 epidemic: an observational cohort study. Lancet. 2020;395(10239):1771-1778. doi:10.1016/S0140-6736(20)31103-X 32410760 PMC7220177

[4] Toubiana J, Poirault C, Corsia A, . Kawasaki-like multisystem inflammatory syndrome in children during the COVID-19 pandemic in Paris, France: prospective observational study. BMJ. 2020;369:m2094. doi:10.1136/bmj.m2094 32493739 PMC7500538

[5] Lee PY, Day-Lewis M, Henderson LA, . Distinct clinical and immunological features of SARS-CoV-2–induced multisystem inflammatory syndrome in children. J Clin Invest. 2020;130(11):5942-5950. doi:10.1172/JCI141113 32701511 PMC7598077

[6] Corwin DJ, Sartori LF, Chiotos K, . Distinguishing multisystem inflammatory syndrome in children from Kawasaki disease and benign inflammatory illnesses in the SARS-CoV-2 pandemic. Pediatr Emerg Care. 2020;36(11):554-558. doi:10.1097/PEC.0000000000002248 32970023 PMC8555855

[7] Jonat B, Gorelik M, Boneparth A, . Multisystem inflammatory syndrome in children associated with coronavirus disease 2019 in a children’s hospital in New York City: patient characteristics and an institutional protocol for evaluation, management, and follow-up. Pediatr Crit Care Med. 2021;22(3):e178-e191. doi:10.1097/PCC.0000000000002598 33003176 PMC7924927

[8] Henderson LA, Canna SW, Friedman KG, . American College of Rheumatology clinical guidance for multisystem inflammatory syndrome in children associated with SARS-CoV-2 and hyperinflammation in pediatric COVID-19: Version 3. Arthritis Rheumatol. 2022;74(4):e1-e20. doi:10.1002/art.42062 35118829 PMC9011620

[9] Harwood R, Allin B, Jones CE, ; PIMS-TS National Consensus Management Study Group. A national consensus management pathway for paediatric inflammatory multisystem syndrome temporally associated with COVID-19 (PIMS-TS): results of a national Delphi process. Lancet Child Adolesc Health. 2021;5(2):133-141. doi:10.1016/S2352-4642(20)30304-7 32956615 PMC7500943

[10] Newburger JW, Takahashi M, Burns JC, . The treatment of Kawasaki syndrome with intravenous gamma globulin. N Engl J Med. 1986;315(6):341-347. doi:10.1056/NEJM198608073150601 2426590

[11] Kobayashi T, Saji T, Otani T, ; RAISE study group investigators. Efficacy of immunoglobulin plus prednisolone for prevention of coronary artery abnormalities in severe Kawasaki disease (RAISE study): a randomised, open-label, blinded-endpoints trial. Lancet. 2012;379(9826):1613-1620. doi:10.1016/S0140-6736(11)61930-2 22405251

[12] Son MBF, Murray N, Friedman K, ; Overcoming COVID-19 Investigators. Multisystem inflammatory syndrome in children—initial therapy and outcomes. N Engl J Med. 2021;385(1):23-34. doi:10.1056/NEJMoa2102605 34133855 PMC8220972

[13] Ouldali N, Toubiana J, Antona D, ; French Covid-19 Paediatric Inflammation Consortium. Association of intravenous immunoglobulins plus methylprednisolone vs immunoglobulins alone with course of fever in multisystem inflammatory syndrome in children. JAMA. 2021;325(9):855-864. doi:10.1001/jama.2021.0694 33523115 PMC7851757

[14] McArdle AJ, Vito O, Patel H, ; BATS Consortium. Treatment of multisystem inflammatory syndrome in children. N Engl J Med. 2021;385(1):11-22. doi:10.1056/NEJMoa2102968 34133854 PMC8220965

[15] Villacis-Nunez DS, Jones K, Jabbar A, . Short-term outcomes of corticosteroid monotherapy in multisystem inflammatory syndrome in children. JAMA Pediatr. 2022;176(6):576-584. doi:10.1001/jamapediatrics.2022.0292 PMC8961405 35344042

[16] Mahmoud S, El-Kalliny M, Kotby A, El-Ganzoury M, Fouda E, Ibrahim H. Treatment of MIS-C in children and adolescents. Curr Pediatr Rep. 2022;10(1):1-10. doi:10.1007/s40124-021-00259-4 35036079 PMC8741532

[17] Chou J, Thomas PG, Randolph AG. Immunology of SARS-CoV-2 infection in children. Nat Immunol. 2022;23(2):177-185. doi:10.1038/s41590-021-01123-9 35105983 PMC8981222

[18] Kobayashi T, Kobayashi T, Morikawa A, . Efficacy of intravenous immunoglobulin combined with prednisolone following resistance to initial intravenous immunoglobulin treatment of acute Kawasaki disease. J Pediatr. 2013;163(2):521-526. doi:10.1016/j.jpeds.2013.01.022 23485027

[19] Weiss PF, Feinstein JA, Luan X, Burnham JM, Feudtner C. Effects of corticosteroid on Henoch-Schönlein purpura: a systematic review. Pediatrics. 2007;120(5):1079-1087. doi:10.1542/peds.2007-0667 17974746 PMC3525094

[20] Leung AKC, Barankin B, Leong KF. Henoch-Schönlein purpura in children: an updated review. Curr Pediatr Rev. 2020;16(4):265-276. doi:10.2174/18756336MTA2lNDYc2 32384035

[21] Aljebab F, Choonara I, Conroy S. Systematic review of the toxicity of long-course oral corticosteroids in children. PLoS One. 2017;12(1):e0170259. doi:10.1371/journal.pone.0170259 28125632 PMC5268779

[22] von Elm E, Altman DG, Egger M, Pocock SJ, Gøtzsche PC, Vandenbroucke JP; STROBE Initiative. The Strengthening the Reporting of Observational Studies in Epidemiology (STROBE) statement: guidelines for reporting observational studies. J Clin Epidemiol. 2008;61(4):344-349. doi:10.1016/j.jclinepi.2007.11.008 18313558

[23] Electronic Code of Federal Regulations Part 46—Protection of Human Subjects. 2017. Federal Policy for the Protection of Human Subjects. Accessed October 7, 2022. https://www.federalregister.gov/documents/2017/01/19/2017-01058/federal-policy-for-the-protection-of-human-subjects

[24] Novelli L, Motta F, De Santis M, Ansari AA, Gershwin ME, Selmi C. The JANUS of chronic inflammatory and autoimmune diseases onset during COVID-19—a systematic review of the literature. J Autoimmun. 2021;117:102592. doi:10.1016/j.jaut.2020.102592 33401171 PMC7833462

[25] Lee PY, Platt CD, Weeks S, . Immune dysregulation and multisystem inflammatory syndrome in children (MIS-C) in individuals with haploinsufficiency of SOCS1. J Allergy Clin Immunol. 2020;146(5):1194-1200.e1. doi:10.1016/j.jaci.2020.07.033 32853638 PMC7445138

[26] Bhattacharjee S, Banerjee M. Immune thrombocytopenia secondary to COVID-19: a systematic review. SN Compr Clin Med. 2020;2(11):2048-2058. doi:10.1007/s42399-020-00521-8 32984764 PMC7501509

[27] Barrett CE, Koyama AK, Alvarez P, . Risk for newly diagnosed diabetes >30 days after SARS-CoV-2 infection among persons aged <18 years—United States, March 1, 2020-June 28, 2021. MMWR Morb Mortal Wkly Rep. 2022;71(2):59-65. doi:10.15585/mmwr.mm7102e2 35025851 PMC8757617

[28] Truong DT, Trachtenberg FL, Pearson GD, ; MUSIC Study Investigators (Supplement 1). The NHLBI Study on Long-terM OUtcomes after the Multisystem Inflammatory Syndrome In Children (MUSIC): Design and Objectives. Am Heart J. 2022;243:43-53. doi:10.1016/j.ahj.2021.08.003 34418362 PMC8710361

[29] Centers for Disease Control and Prevention. BMI percentile calculator for child and teen. 2022. Accessed April 27, 2022. https://www.cdc.gov/healthyweight/bmi/calculator.html

[30] Feldstein LR, Tenforde MW, Friedman KG, ; Overcoming COVID-19 Investigators. Characteristics and outcomes of US children and adolescents with multisystem inflammatory syndrome in children (MIS-C) compared with severe acute COVID-19. JAMA. 2021;325(11):1074-1087. doi:10.1001/jama.2021.2091 33625505 PMC7905703

[31] World Health Organization. Clinical management of COVID-19 patients: living guideline. September 14, 2022. Accessed October 7, 2022. https://app.magicapp.org/#/guideline/j1WBYn

[32] Multisystem inflammatory syndrome therapies in children (MISTIC) comparative effectiveness study. April 6, 2022. Accessed September 15, 2022. https://clinicaltrials.gov/ct2/show/NCT04898231

[33] Ringold S, Nigrovic PA, Feldman BM, . The Childhood Arthritis and Rheumatology Research Alliance Consensus treatment plans: toward comparative effectiveness in the pediatric rheumatic diseases. Arthritis Rheumatol. 2018;70(5):669-678. doi:10.1002/art.40395 29333701

[34] Levy N, Koppel JH, Kaplan O, . Severity and incidence of multisystem inflammatory syndrome in children during 3 SARS-CoV-2 pandemic waves in Israel. JAMA. 2022;327(24):2452-2454. doi:10.1001/jama.2022.8025 35588048 PMC9121298

[35] Cloete J, Kruger A, Masha M, . Paediatric hospitalisations due to COVID-19 during the first SARS-CoV-2 Omicron (B.1.1.529) variant wave in South Africa: a multicentre observational study. Lancet Child Adolesc Health. 2022;6(5):294-302. doi:10.1016/S2352-4642(22)00027-X 35189083 PMC8856663

[36] Holm M, Espenhain L, Glenthøj J, . Risk and phenotype of multisystem inflammatory syndrome in vaccinated and unvaccinated Danish children before and during the Omicron wave. JAMA Pediatr. 2022;176(8):821-823. doi:10.1001/jamapediatrics.2022.2206 35675054 PMC9178498

[37] Srinivasan V. Stress hyperglycemia in pediatric critical illness: the intensive care unit adds to the stress! J Diabetes Sci Technol. 2012;6(1):37-47. doi:10.1177/193229681200600106 22401321 PMC3320820

[38] Tillotson CV, Bowden SA, Boktor SW. Pediatric type 2 diabetes mellitus. StatPearls Publishing; 2022. 28613700

[39] Dufort EM, Koumans EH, Chow EJ, ; New York State and Centers for Disease Control and Prevention Multisystem Inflammatory Syndrome in Children Investigation Team. Multisystem inflammatory syndrome in children in New York State. N Engl J Med. 2020;383(4):347-358. doi:10.1056/NEJMoa2021756 32598830 PMC7346766

[40] Whittaker E, Bamford A, Kenny J, ; PIMS-TS Study Group and EUCLIDS and PERFORM Consortia. Clinical characteristics of 58 children with a pediatric inflammatory multisystem syndrome temporally associated with SARS-CoV-2. JAMA. 2020;324(3):259-269. doi:10.1001/jama.2020.10369 32511692 PMC7281356

