# Supplementary material for: Postdischarge Glucocorticoid Use and Clinical Outcomes of Multisystem Inflammatory Syndrome in Children
Source: JAMA Netw Open. 2022 Nov 11;5(11):e2241622. doi: 10.1001/jamanetworkopen.2022.41622 (PMC9652757; doi:10.1001/jamanetworkopen.2022.41622)
Supplement: Supplement 1. — eTable 1. Glucocorticoid Inpatient Regimens and Outcomes for Patients Treated with Postdischarge Glucocorticoids eTable 2. Demographic and Clinical Characteristics of Patients Based on Outpatient Immunomodulatory Treatment eTable 3. Inpatient Immunomodulatory Treatment Regimens eFigure 1. Distribution of Patients Across Participating Centers and According to Duration of Glucocorticoid Outpatient Regimen eFigure 2. C-Reactive Protein (CRP) Measurements per Patient and Ferritin Measurements per Patient, According to Duration of Outpatient Glucocorticoid Regimen, in the Six Weeks Following Hospital Discharge [file jamanetwopen-e2241622-s001.pdf]

Supplementary Online Content

Son MBF, Berbert L, Young C, et al. Postdischarge glucocorticoid use and clinical outcomes of multisystem inflammatory syndrome in children. *JAMA Netw Open*. 2022;5(11):e2241622. doi:10.1001/jamanetworkopen.2022.41622

eTable 1. Glucocorticoid Inpatient Regimens and Outcomes for Patients Treated with Postdischarge Glucocorticoids

eTable 2. Demographic and Clinical Characteristics of Patients Based on Outpatient Immunomodulatory Treatment

eTable 3. Inpatient Immunomodulatory Treatment Regimens

eFigure 1. Distribution of Patients Across Participating Centers and According to Duration of Glucocorticoid Outpatient Regimen

eFigure 2. C-Reactive Protein (CRP) Measurements per Patient and Ferritin Measurements per Patient, According to Duration of Outpatient Glucocorticoid Regimen, in the Six Weeks Following Hospital Discharge

This supplementary material has been provided by the authors to give readers additional information about their work.

© 2022 Son MBF et al. *JAMA Network Open.*

eTable 1: Glucocorticoid Inpatient Regimens and Outcomes for Patients Treated with Postdischarge Glucocorticoids[a]

Inpatient Intravenous Glucocorticoid Dosing	Inpatient Oral Glucocorticoid Dosing	N (%)	Gained ≥2 kg in 90 days post discharge n (%)	Hyperglycemia (serum glucose ≥250 mg/dL) n, (%)
None or Low Dose	None or Low Dose	50 (28.9%)	17 (22.7%)	3 (21.4%)
None or Low Dose	High Dose	67 (38.7%)	32 (42.7%)	5 (35.7%)
High Dose	None or Low Dose	33 (19.1%)	8 (10.7%)	2 (14.3%)
High Dose	High Dose	23 (13.3%)	18 (24%)	4 (28.6%)

[a] For inpatient intravenous glucocorticoids, high was dose defined as ≥10mg/kg/day or ≥500 mg/day. For inpatient oral glucocorticoids, high dose was defined as ≥2mg/kg/day or ≥60 mg/day. Two patients received no IV and no oral glucocorticoids, two received no IV glucocorticoids but received oral, 25 received IV but not oral glucocorticoids, and the remainder (n=145) received both.

© 2022 Son MBF et al. *JAMA Network Open.*

eTable 2: Demographic and Clinical Characteristics of Patients Based on Outpatient Immunomodulatory Treatment

	All (n=186)	Prescribed Outpatient Immunomodulatory Treatment (n=174)	No Outpatient Immunomodulatory Treatment (n=12)
Age (median, IQR)	10.4 (6.7, 14.2)	10.2 (6.2, 13.8)	14.4 (10.7, 15.5)[a]
Age (n,%)			
• < 1 year	3 (1.6%)	3 (1.7%)	0 (0%)
• 1-4 years	28 (15.1%)	28 (16.1%)	0 (0%)
• 5-9 years	56 (30.1%)	53 (30.5%)	3 (25%)
• 10-14 years	67 (36%)	63 (36.2%)	4 (33.3%)
• 15-20 years	32 (17%)	27 (15.5%)	5 (41.7%)
Male Sex (n,%)	107 (57.5%)	98 (56.3%)	9 (75%)
Female Sex (n,%)	79 (42.5%)	76 (43.7%)	3 (25%)
Race/Ethnicity (n,%)			
• Asian	3 (1.6%)	3 (1.7%)	0 (0%)
• Black, non-Hispanic	60 (32.3%)	53 (30.5%)	7 (58.3%)
• Hispanic or Latino	59 (31.7%)	55 (31.6%)	4 (33.3%)
• White, non-Hispanic	40 (21.5%)	40 (23%)	0 (0%)
• Other, non-Hispanic[b]	8 (4.3%)	8 (4.6%)	0 (0%)
• Unknown	16 (8.6%)	15 (8.6%)	1 (8.3%)
BMI based obesity n=184 (n,%)	60 (32.6%)	56 (32.6%)	4 (33.3%)
Not previously healthy (n=185) [c]	49 (26.5%)	44 (25.4%)	5 (41.7%)
# of Organ Systems Involved			
• ≤4	61 (32.8%)	59 (33.9%)	2 (16.7%)
• ≥5	125 (67.8%)	115 (66.1%)	10 (83.3%)

© 2022 Son MBF et al. *JAMA Network Open.*

	All (n=186)	Prescribed Outpatient Immunomodulatory Treatment (n=174)	No Outpatient Immunomodulatory Treatment (n=12)
Met Criteria for Incomplete or Complete Kawasaki Disease (n,%)	63 (33.9%)	60 (34.5%)	3 (25.0%)
Median Days of Fever n= 166 (median, IQR, range)	5 [4, 6] (0, 23)	5 [4, 6] (0, 23)	5 [3.5, 7.2] (0, 18)
ICU admission n=186 (n,%)	163 (87.6%)	152 (87.4%)	11 (91.7%)
Median Length of ICU Stay n=163 (median, IQR, range)	3 [2, 5] (0, 26)	3 [2, 5] (0, 26)	4 [2.5, 5.5] (1, 26)
Median Length of Hospital Stay n=186 (median, IQR, range)	7 [5, 9] (1, 38)	7 [5, 9] (1, 36)	7 [5.8, 10.5] (3, 38)
Supplemental Oxygen n=185 (n,%)	133 (71.9%)	124 (71.7%)	9 (75%)
Mechanical Ventilation n=181 (n,%)	29 (15.7%)	26 (15%)	3 (25%)
Vasopressor Requirement n=186 (n,%)	134 (72%)	126 (72.4%)	8 (66.7%)
ECMO n=179 (n,%)	5 (2.7%)	4 (2.3%)	1 (8.3%)

[a]p=0.014

[b] Other, non-Hispanic included: Alaskan Native, American Indian, Native Hawaiian, Pacific Islander and other.

[c]Not previously healthy was defined as the presence of reported underlying conditions (respiratory, cardiovascular, neurologic, oncologic, immunosuppressive, rheumatologic or autoimmune, hematologic, kidney or urologic, gastrointestinal or hepatic, endocrine, or metabolic [including obesity]); long-term ventilation or oxygen support; and use of prescription diuretics, bronchodilators, glucocorticoids, statins, immunosuppressive drugs, or chemotherapy for any condition.

© 2022 Son MBF et al. *JAMA Network Open.*

eTable 3: Inpatient Immunomodulatory Treatment Regimens

**Intravenous Immunoglobulin (n=186)**	
First Dose (n=186, 100%):<br>• Day of hospitalization administered: n [IQR] (range)<br>• Dose (g/kg)<br>Second Dose (n=26, 14%):<br>• Day of hospitalization administered: n [IQR] (range)<br>• Dose (g/kg)	• 1 [0,1] (0,9)<br>• 2 [1.7, 2] (0.6, 2.1)<br><br>• 2.5 [2, 3.8) (0, 12)<br>• 1 [1, 2] (0.5, 2)
**Intravenous glucocorticoids (n=178)[a]**	
Methylprednisolone (n=173, 97%)<br>• Day of hospitalization: n [IQR] (range)<br>• Dose (maximum) mg/kg/day (n=159)<br>• Dose (maximum) mg/day (n=171)<br>• Number of days prescribed	• 1 [0, 1] (0, 22)<br>• 2 [1.9, 10] (0.5, 33.3)<br>• 100 [60, 500] (12.4, 1000.8)<br>• 4 [3, 6] (1, 23)
Dexamethasone (n=3, 1.7%)<br>• Day of hospitalization: n [IQR] (range)<br>• Dose (maximum) mg/kg/day<br>• Dose (maximum) mg/day<br>• Number of days prescribed	• 1 [1,2] (0,3)<br>• 0.5 [0.1, 2] (0.1, 7.5)<br>• 6 [6, 23] (6, 500)<br>• 4 [3, 4] (1, 10)
**Oral glucocorticoids (n=151)[b]**	
Prednisone (n=79, 52%)<br>• Day of hospitalization: n [IQR] (range)<br>• Dose (maximum) mg/kg/day (n=77)<br>• Dose (maximum) mg/day (n=79)<br>• Number of days prescribed (n=78)<br>• Dose at discharge mg/kg/day (n=74)<br>• Dose at discharge mg/day (n=76)	• 5 [4, 7] (0, 29)<br>• 1 [0.8, 1.7] (0.2, 2.3)<br>• 60 [45, 80] (10, 121)<br>• 2.5 [2, 3] (1, 9)<br>• 1 [0.7, 1.4] (0.1, 2.3)<br>• 60 [40, 60] (5, 120)
Methylprednisolone (n=70, 46.4%)<br>• Day of hospitalization: n [IQR] (range)<br>• Dose (maximum) mg/kg/day (n=69)<br>• Dose (maximum) mg/day (n=70)<br>• Number of days prescribed (n=69)<br>• Dose at discharge mg/kg/day (n=66)<br>• Dose at discharge mg/day (n=67)	• 4 [3, 6.8] (0, 23)<br>• 1.6 [1, 2] (0.1, 3.6)<br>• 36.6 [27.4, 54.7] (2, 80)<br>• 2 [2, 3] (1, 22)<br>• 1.6 [1, 2] (0.1, 2.5)<br>• 36 [24, 52.4] (2, 80)
**Anakinra (n=47, IV or SubQ)**	
• Day of hospitalization: n [IQR] (range)<br>• Dose (maximum) mg/kg/day (n=46)<br>• Dose (maximum) mg/day<br>• Number of days prescribed<br>• Dose at discharge (n=9) mg/day	• 1 [1, 3] (0, 11)<br>• 4.4 [3.2, 5.6] (0.7, 10.8)<br>• 300 [200, 400] (22.4, 400)<br>• 8 [6, 13] (2, 27)<br>• 200 (100, 200) [25, 400]

© 2022 Son MBF et al. *JAMA Network Open.*

**TNF inhibitors (n=1)**	
• Medication<br>○ Etanercept (n=1)	○ 0.8 mg/kg/dose x 1 dose

[a]2 patients received both methylprednisolone and dexamethasone
[b]2 patients received oral dexamethasone

© 2022 Son MBF et al. *JAMA Network Open.*

<u>eFigure 1:</u> Distribution of patients across participating centers* and according to duration of glucocorticoid outpatient regimen

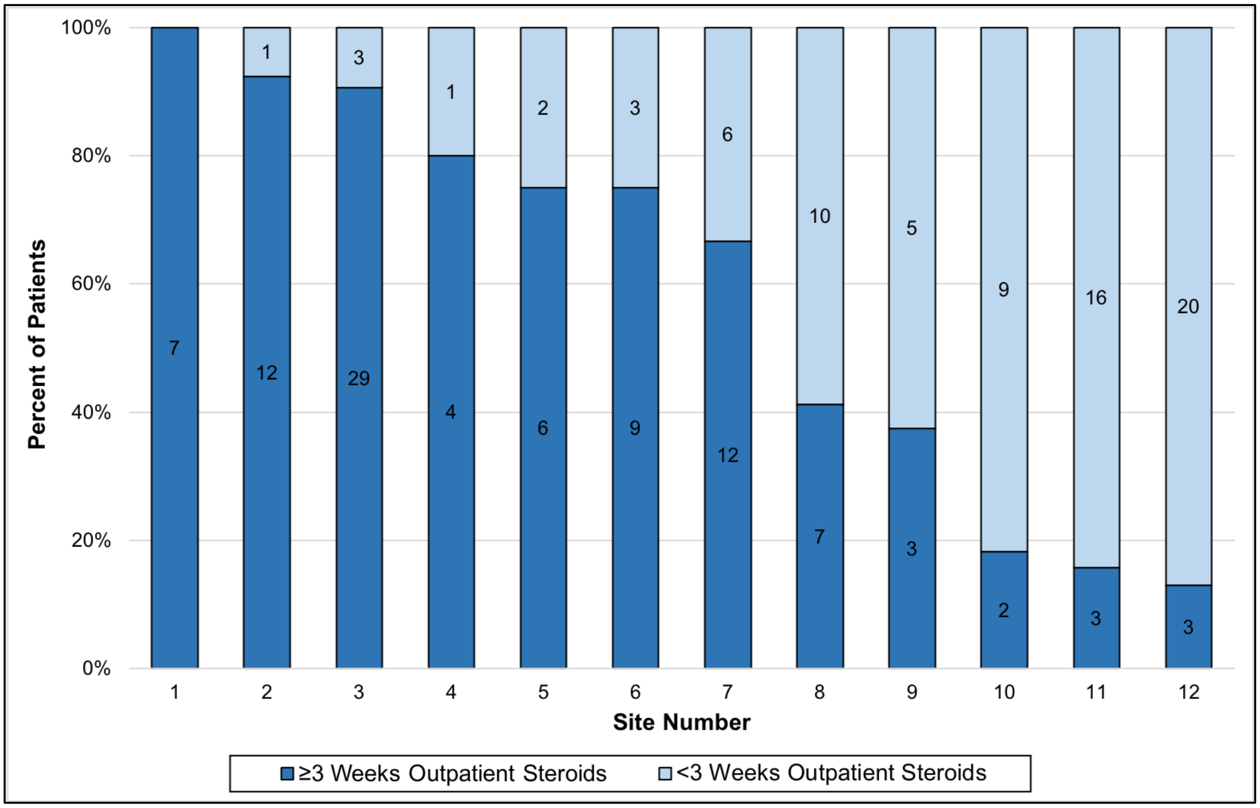

[a]One site did not prescribe post-discharge glucocorticoids

© 2022 Son MBF et al. *JAMA Network Open.*

e<u>Figure 2A:</u> C-reactive protein (CRP) measurements per patient, according to duration of outpatient glucocorticoid regimen, in the six weeks following hospital discharge.

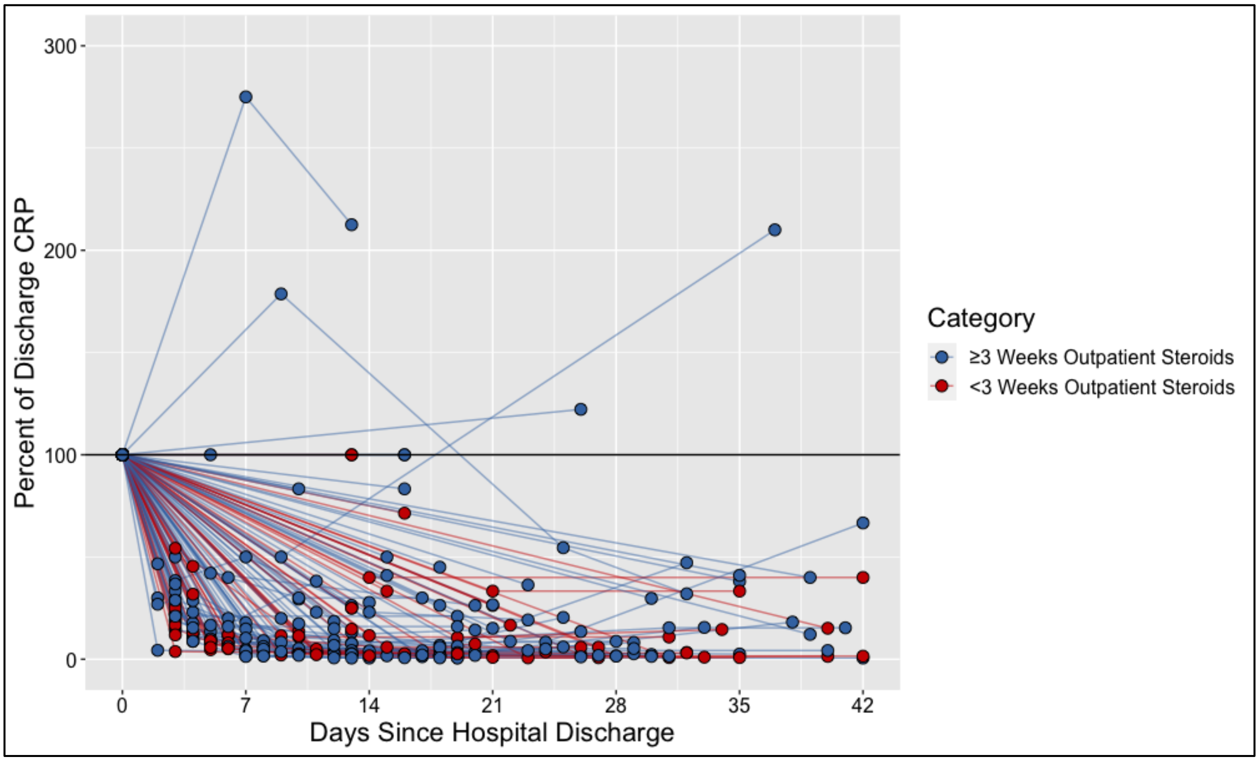

© 2022 Son MBF et al. *JAMA Network Open.*

eFigure 2B: Ferritin measurements per patient, according to duration of outpatient glucocorticoid regimen, in the six weeks following hospital discharge.

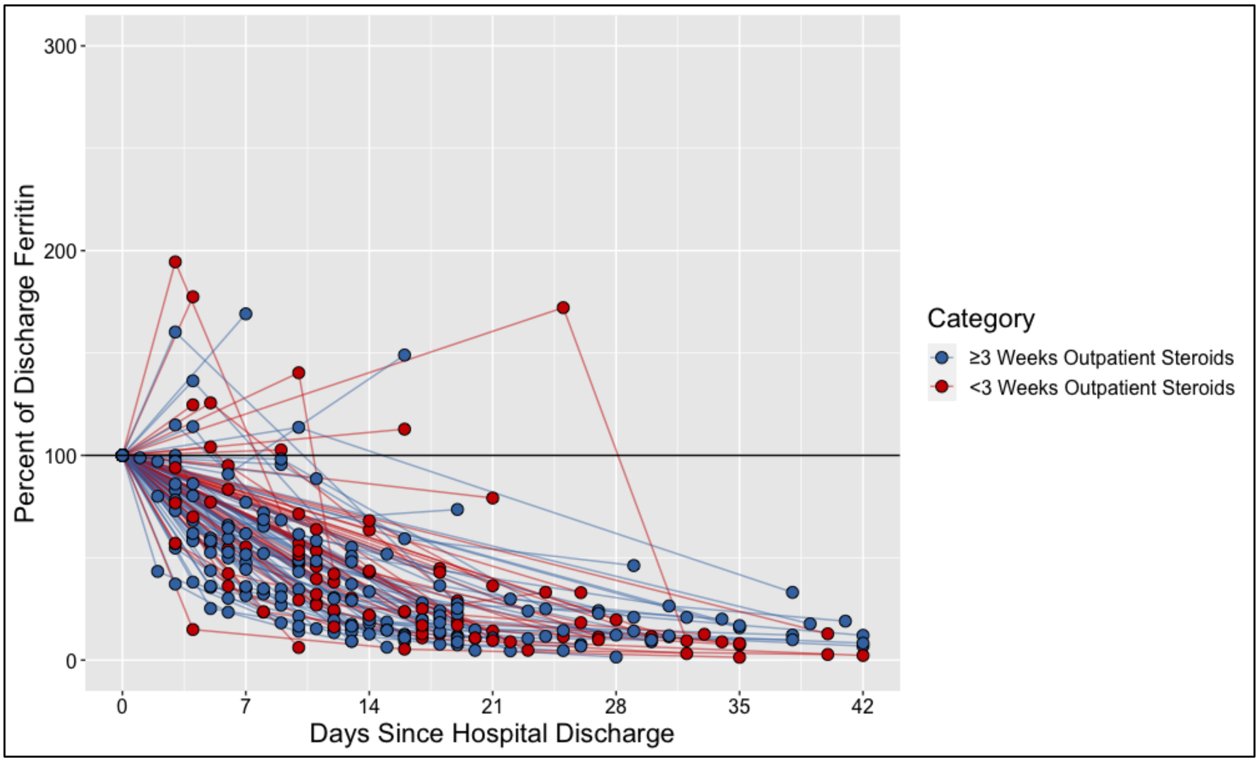

© 2022 Son MBF et al. *JAMA Network Open.*
