# Supplementary material for: Postdischarge Glucocorticoid Use and Clinical Outcomes of Multisystem Inflammatory Syndrome in Children
Source: JAMA Netw Open. 2022 Nov 11;5(11):e2241622. doi: 10.1001/jamanetworkopen.2022.41622 (PMC9652757; doi:10.1001/jamanetworkopen.2022.41622)
Supplement: Supplement 2. — Overcoming COVID-19 Investigators [file jamanetwopen-e2241622-s002.pdf]

Supplemental Online Content: Nonauthor Collaborators
*First name, last name, and suffix (if applicable) are required and will appear in PubMed.

*Group Name(s): Overcoming COVID-19 Investigators							
***First Name and Middle Initial(s)**	***Last Name**	***Suffix (eg, Jr, III)**	Academic Degrees	Institution	Location (city, state/province, country)	Role or Contribution, eg, chair, principal investigator	Group (if more than 1 Group listed in the byline) and/or Subgroup (eg, Steering Committee)
Michele	Kong						
Natalie	Cvijanovich						
Matt S	Zinter						
Keiko M	Tarquinio						
Suden	Kucukak						
Madyson M	FitzGerald						
Julie	Worden						
Emily R	Levy						
Lora	Martin						
Lacy	Malloch						
Katharine N	Clouser						
Stephanie P	Schwartz						
Tracie C	Walker						
Mark W	Hall						
Mary A	Staat						
Laura L	Loftis						
Lincoln S	Smith						
John K	McGuire						
Leora R	Feldstein						
Mark W	Tenforde						
Ashley M	Jackson						
